# Supplementary material for: The climate impact and land use of cultivated meat: Evaluating agricultural feedstock production
Source: PLoS One. 2025 Jan 22;20(1):e0316480. doi: 10.1371/journal.pone.0316480 (PMC11753643; doi:10.1371/journal.pone.0316480)
Supplement: Attachment — Submitted filename: Response to Reviewers.docx [file pone.0316480.s001.docx]

| PLOS ONE | | |
| --- | --- | --- |
| **The Climate Impact and Land Use of Cultivated Meat: Evaluating Agricultural Feedstock Production - PONE-D-24-43823** | | |
| **Reviewers‘ Comments** | **Comments** | **Page** |
| Reviewer 1 |  |  |
| In the introductions of each section, such as the Background of CM and Materials and Methods, I suggest beginning by clearly identifying what will be described. Below, I have provided an example of how this can be adjusted as a suggestion. - In the Background of CM: "This section outlines" could be changed to "Technical and environmental aspects of MC production, innovative approaches, regulatory challenges, and the importance of transparent and accurate information for consumer acceptance will be described." - In Methods and Scenarios, I suggest starting directly with the description of items 3.1, 3.2, and 3.3 without an introductory text, as each subsection will play the role of explaining and contextualizing the content. | Thank you for your valuable feedback. We have revised the introductions for each section, as suggested, to clearly identify the content that follows. In the Background of CM, we now specify the technical and environmental aspects, innovative approaches, regulatory challenges, and the importance of transparency in consumer acceptance. For Methods and Scenarios, we incorporated brief, direct introductions in each subsection (3.1, 3.2, and 3.3) to effectively contextualize the content without additional introductory text. We believe these adjustments enhance the clarity and flow as recommended. | 5 |
| In Table 1: - Clarify in the caption what (t/ha) stands for. | We have revised the caption for Table 1 to clarify that yields are presented in tons per hectare (t/ha) as suggested. The updated caption now reads: *"Average crop yields (in tons per hectare, t/ha) in southern Germany, Germany, the world, and continents in 2021."* This addition should improve clarity for readers. | 16 |
| In line 412 and line 421: - I suggest removing the word "see" from the expressions (see Table 2) and (see Table 3), as it is unnecessary; simply (Table 2) and (Table 3) are sufficient. | Thank you for the suggestion. We have removed the word "see" from the expressions in lines 412 and 421, so they now read simply as "(Table 2)" and "(Table 3)" to improve conciseness. | 17 |
| Clarify what the asterisk (*) means, as referenced in line 440. | Thank you for pointing this out. We have replaced the asterisk (*) with a multiplication symbol (×) in line 440 to enhance clarity. This adjustment should make the notation more straightforward for readers. | 17 |
| In Table 3: - Explain what "wm" refers to in "g/g CM wm stoich. Demand" in the caption. | We have updated the caption for Table 3 to clarify the abbreviations. It now reads: "Relative nutrient requirements for cell mass production g/g CM = gram per gram cultivated meat; FM = Fresh mass)." This should improve clarity for readers. | 19 |
| Provide an explanation or reference for what the "INRAE-CIRAD-AFZ" table refers to. | Thank you for the suggestion. We have added an explanation for the INRAE-CIRAD-AFZ Feed Tables, clarifying its purpose and origin. Additionally, we noticed a duplication of the sentence below Table 3 and have removed it for conciseness. | 17 |
| In Table 4: - I suggest formatting the text using the same font color; if you'd like to highlight something, use bold. - Additionally, the borders should be adjusted to match in color, line thickness, and formatting, consistent with the other tables. - All text should start with an uppercase letter. - Include in the caption what DM stands for. - If words are underlined, I recommend standardizing them. If you want to provide information on these underlined items, include them in plain text. | Thank you for the detailed feedback. We have implemented the suggested changes in Table 4 by standardizing to black font color with bold for emphasis, adjusting border color, line thickness, and formatting to match the other tables. We have also ensured all text begins with uppercase letters, spelled out “DM”, and removed any underlining for consistency. These adjustments should enhance readability and uniformity across the tables. | 21 |
| In lines 478 to 481: - There are duplicate texts; I suggest removing them. | Thank you for catching that oversight. We have removed the duplicate text in lines 478 to 481 as suggested. | 22 |
| In Table 5: - Standardize the words within parentheses by capitalizing the first letter in all lines. - Explain these units of measurement in the caption: t FM/ha. - I suggest removing "corr. Ø" and explaining these items clearly. | Thank you for the helpful comments. We have standardized the capitalization within parentheses in Table 5. Additionally, we clarified the units of measurement in the caption, explaining "t FM/ha" as tons per hectare of fresh matter, and rephrased to remove "corr. Ø," providing a clear explanation of adjusted average yields. | 22 |
| In Table 6: Follow the same standardization as the previous tables. - I suggest formatting the text using the same font color; if you'd like to highlight something, use bold. - Additionally, the borders should be adjusted to match in color, line thickness, and formatting, consistent with the other tables. - All text should start with an uppercase letter. - If words are underlined, I recommend standardizing them. If you want to provide information on these underlined items, include them in plain text. | Thank you for your feedback. We have applied the same standardization as in previous tables by using a single font color with bold for emphasis, adjusting borders for consistency, capitalizing all text at the start, and removing underlining. | 23 |
| In Table 7: - Clarify in the caption what LCA stands for. | Thank you for the suggestion. We have clarified in the caption that "LCA" stands for Life Cycle Assessment to improve understanding for readers. | 29 |
| I suggest adding a separate section on limitations and future perspectives, which would bring clarity and flow to the text. | We separated the “Discussion” section into three parts to improve clarity and flow. | 34 |
| I recommend discussing a few important points, such as: - In addition to production and environmental costs, where would the financial costs fit in? Where would the funding come from? - In this study, was there specific funding for obtaining the raw materials? If so, I suggest describing it. - For more extensive treatments in this production, how would the product’s purity level be achieved? - Regarding the practical viability of large-scale cultured meat production, including considerations of cost and resource demand, I suggest discussing these aspects. | Thank you for your valuable feedback. We have addressed your points as follows:   - **Financial Costs and Funding Sources:** We have added a discussion on the economic aspects of cultivated meat (CM) production, highlighting that the high cost of growth media is a significant financial barrier. This includes the challenges of funding and the need for cost-effective solutions to make CM competitive with conventional meat. Details are included in the revised “2.3 Economic viability and impacts on the meat industry and farming communities” section and in the “5.2 Limitations” section. - **Funding for Raw Materials:** Our study is a theoretical modeling analysis that utilized existing data, so there was no need for specific funding to obtain raw materials. We have clarified this in the "Methods and Scenarios" section and expanded the "Acknowledgements" to describe the funding that supported our research. - **Achieving Product Purity:** We have included a discussion on the challenges of achieving the necessary purity levels for amino acids and other nutrients in large-scale CM production. This addresses the technical and cost implications of refining crop-based substrates to meet bioreactor requirements, as detailed in the "5.1 Comparison with existing literature" section. | 10/35/36 |
| In Acknowledgements: - You mention that funding for research was provided by the Bavarian State Government as part of the Tech Agenda Bavaria. - What specifically was this funding for? I suggest describing this in the Methods section and also updating the initial checklist where it is declared that no funding was received. | We added a clearer explanation to the funding obtained:  “Funding for the author's research position, which supported the completion of this study, was provided by the Bavarian State Government as part of the High-Tech Agenda Bavaria.” | 52 |
| Reviewer 2 |  |  |
| While the study acknowledges the role of solar energy and the high energy demands of CM production, it seems to underemphasize the broader energy costs involved in cultivating meat. The focus on land use efficiency might obscure the significant environmental footprint associated with energy consumption in these production systems, especially as the energy sources transition from fossil fuels to renewables. | Thank you for highlighting the need to address the broader energy costs in cultivated meat (CM) production. We agree that focusing solely on land use efficiency may overlook the significant environmental footprint and cost associated with the high energy demands of CM, especially during the transition from fossil fuels to renewable energy sources.  To address this, we have added a brief discussion in the manuscript under the “Limitations” section 5.2. | 37 |
| The study rightly considers crop rotation, but it doesn't explore more innovative or regenerative agricultural practices that could reduce land requirements or enhance sustainability. This should be considered in the discussion. | Thank you for your valuable suggestion regarding the exploration of innovative and regenerative agricultural practices. We agree that considering these practices could provide additional insights into reducing land requirements and enhancing sustainability in cultivated meat (CM) production.  To address this, we have added a discussion in the manuscript under the "5.2 Limitations” section:  "Furthermore, our data was based on German yield averages. Future integrated and regenerative agricultural practices could lead to different results, although lower crop yields and therefore lower sugar and amino acid yields are to be expected with these cultivation methods [113]. Moreover, conservation agriculture does not generally lead to N₂O emission mitigation [114]." | 36 |
| There is no discussion of the economic feasibility of CM production using agricultural feedstock. While the study focuses on land use, understanding the real financial costs would be essential for assessing the viability of these studies. | We addressed your comment, in the newly added “5.2 Limitations” section. In this section, we discuss the significant financial challenges associated with CM production:   - **High Cost of Growth Media:** We highlight that the cost of growth media remains a major financial bottleneck in CM production. Current estimates indicate that media expenses can account for up to 99% of material costs, primarily due to the expensive amino acids and growth factors produced through precision fermentation or chemical synthesis [37,41,115]. - **Challenges with Precision Fermentation:** While precision fermentation can reduce land use impacts, its complexity and high production costs pose significant challenges. The production of growth factors through chemical synthesis adds to the economic burden [37,115]. - **Potential of Plant-Based Media:** Emerging research suggests that plant-based media could offer a more cost-effective solution. However, this remains speculative as the feasibility of using hydrolysates for cell media formulation has not been fully established [37,115]. - **Scaling Bioreactors:** Even as bioreactor scales increase, reducing media costs is critical to achieving competitive prices with conventional meat [116]. | 36 |
| The study touches on climate change mitigation but does not provide a detailed analysis of other environmental factors such as water use, biodiversity, or soil health impacts from agricultural feedstock use. CM's sustainability should be evaluated through a broader lens of environmental indicators. | Thank you for pointing out the need to evaluate cultivated meat (CM) sustainability through a broader range of environmental indicators beyond land use. We agree that factors such as water use, biodiversity, and soil health are essential components of a comprehensive assessment.  We addressed this in the end of the “5.2 Limitations” section: “Furthermore, the model considers only land use and does not account for other critical environmental impacts, such as water usage, influence on biodiversity, or ecosystem structures.”  And in the “5.3 Future research directions” section:  " Future research could build upon the findings of this study by examining the environmental impacts of CM in a more comprehensive and holistic manner, such as by incorporating a range of sustainability metrics beyond land use and energy consumption. To examine CMs full sustainability impacts this will be crucial. This could include additional factors, such as water use, biodiversity impacts, and further potential for carbon sequestration." | 36/38 |
| While the study recommends shifting towards precision fermentation, it doesn't provide enough detail on the challenges or opportunities of adopting this technology. A more nuanced exploration of how precision fermentation and other biotechnological advancements can improve the sustainability of CM and what barriers (technical, regulatory, economic) need to be overcome would make the study more actionable. | Thank you. To address your comment, we have expanded the "5.1 Comparison with existing literature" section to include a detailed analysis of the challenges and opportunities associated with adopting precision fermentation.   - **Technical Challenges:** We highlight the complexities in scaling up precision fermentation technologies, including inadequate global fermentation capacity and the difficulties in engineering productive and robust microbial strains [109]. - **Economic Barriers:** We address the high production costs, noting that media expenses can account for up to 99% of material costs due to expensive amino acids and growth factors produced through precision fermentation or chemical synthesis [37,41,115]. - **Regulatory Hurdles:** We discuss the significant regulatory challenges that persist, although they are somewhat less severe than those for CM itself [110]. - **Environmental Considerations:** We acknowledge that fermentation and chemical processes are water-intensive, which complicates sustainability efforts [41]. | 34/35 |
