# Supplementary material for: The climate impact and land use of cultivated meat: Evaluating agricultural feedstock production
Source: PLoS One. 2025 Jan 22;20(1):e0316480. doi: 10.1371/journal.pone.0316480 (PMC11753643; doi:10.1371/journal.pone.0316480)
Supplement: Attachment — Submitted filename: Response to Reviewers.docx [file pone.0316480.s002.docx]

| PLOS ONE | | |
| --- | --- | --- |
| **The Climate Impact and Land Use of Cultivated Meat: Evaluating Agricultural Feedstock Production - PONE-D-24-43823** | | |
| **Reviewers‘ Comments** | **Comments** | **Page** |
| Reviewer 1 |  |  |
| Most of the issues raised have been addressed; however, I suggest some revisions regarding the tables: Table 1. It is acceptable to use "t/ha" alone, but I recommend adding the note: "t/ha: in tons per hectare" below the description of sources (a) and (b). The caption should be placed below the table, not in its title. Table 3. Similarly, in Table 3, these notes should be added below source (a), described under the table, rather than in the title. Table 4. The table still has borders that differ from the others; I suggest adjusting them. Table 5. As with Tables 1 and 3, the notes I suggested for the caption should be added below source (a), described under the table, and not in the title. Table 7. As with Tables 1, 3, and 5, the notes I suggested for the caption should be added below the table, not in its title. | Thank you for your review and valuable suggestions regarding our tables.  We have made the following revisions accordingly:  • **Tables 1, 3, 5, and 7**: We have moved the notes (e.g., “t/ha: tons per hectare”) below the description of sources placing them under the table rather than in the title.  • **Tables 4, 5, and 6**: We have adjusted the borders of Tables 4, 5, and 6 to ensure consistency with the formatting of the other tables. | 16; 19; 21-25; 29 |
| Reviewer 2 |  |  |
| Reviewer #2: After the reviews made by the authors, I believe that the manuscript can be published since the majority of the pointed concerns were answered and modified by the authors. | Thank you. |  |
