# Supplementary material for: The climate impact and land use of cultivated meat: Evaluating agricultural feedstock production
Source: PLoS One. 2025 Jan 22;20(1):e0316480. doi: 10.1371/journal.pone.0316480 (PMC11753643; doi:10.1371/journal.pone.0316480)
Supplement: Attachment — Submitted filename: Response to Reviewers.docx [file pone.0316480.s003.docx]

| PLOS ONE | | |
| --- | --- | --- |
| **The Climate Impact and Land Use of Cultivated Meat: Evaluating Agricultural Feedstock Production - PONE-D-24-43823** | | |
| **Reviewers‘ Comments** | **Comments** | **Page** |
| Reviewer 1 |  |  |
| Most of the issues raised have been addressed; however, I suggest some revisions regarding the tables: Table 1. It is acceptable to use "t/ha" alone, but I recommend adding the note: "t/ha: in tons per hectare" below the description of sources (a) and (b). The caption should be placed below the table, not in its title. Table 3. Similarly, in Table 3, these notes should be added below source (a), described under the table, rather than in the title. Table 4. The table still has borders that differ from the others; I suggest adjusting them. Table 5. As with Tables 1 and 3, the notes I suggested for the caption should be added below source (a), described under the table, and not in the title. Table 7. As with Tables 1, 3, and 5, the notes I suggested for the caption should be added below the table, not in its title. | Thank you for your review and valuable suggestions regarding our tables.  We have made the revisions accordingly. We incorporated your suggestions as outlined. If there is anything you feel we have not fully addressed, could you please clarify what specific changes are still needed?  We added “Note: m²/kg = square meters per kilogram; LCA = Life Cycle Assessment.” to Table 7. | 16; 19; 21-25; 29 |
